# Impact of Pumpkin Seed, Brown Rice, Yellow Pea, and Hemp Seed Proteins on the Physicochemical, Technological, and Sensory Properties of Green Lentil Cookies

**DOI:** 10.3390/foods14091518

**Published:** 2025-04-26

**Authors:** Réka Juhász, Lívia Hajas, Éva Csajbókné Csobod, Zoltán Pálinkás, Margita Szilágyi-Utczás, Csilla Benedek

**Affiliations:** 1 Department of Dietetics and Nutrition Science, Faculty of Health Science, Semmelweis University, Vas Str. 17, 1088 Budapest, Hungary; hermanne.juhasz.reka@semmelweis.hu (R.J.); csajbokne.csobod.eva@semmelweis.hu (É.C.C.); benedek.csilla@semmelweis.hu (C.B.); 2 Centre for Sports Nutrition Science, Hungarian University of Sport Science, Alkotás Str. 42-48, 1123 Budapest, Hungary; palinkas.zoltan@tf.hu (Z.P.); utczas.margita@tf.hu (M.S.-U.)

**Keywords:** cookie, vegan, gluten-free, lentils, plant proteins, inulin

## Abstract

This study explores the potential of some commercially available plant proteins to increase the protein content of gluten free cookies produced from green lentil flour. Isolates from hemp seed, brown rice, yellow pea, and pumpkin seed were investigated. Cookies were additionally enriched with inulin and matcha tea. Products were characterized in terms of physicochemical parameters (e.g., crude protein content, total phenolics and flavonoids, antioxidant activity, and color). Additionally, technological properties including geometry, baking loss, and texture profile were determined, and a sensory profile test was conducted. The replacement of a quarter of lentil flour with plant proteins increased the protein content (control: 12.4% vs. 15.1–20.4%), but suppressed the polyphenol content, resulting in reduced antioxidant capacity (3.13 vs. 2.14–2.69 mmol TE/100 g). The geometry, texture properties, and color of the cookies were affected by all the proteins investigated. The biggest difference was shown in the case of using yellow pea (YP) protein, which showed the highest browning index (YP: 66.36 vs. 42.63–63.45) and spread ratio (8.38 vs. 5.63–6.39) among the samples tested. The sensory attributes of the cookies, such as tea notes, surface homogeneity, crunchiness, and crumbliness, proved to be negatively affected by the plant proteins, which may be a limitation for consumer acceptance.

## 1. Introduction

In parallel with an increasing health-consciousness, people’s interest in more balanced meals is growing. Consumers prefer functional foods that are practical to carry, easy to consume, and have an increased nutritional value at the same time. Among these, high-protein foods are marketed as weight control and muscle building foods, but recently such foods have also been developed for people struggling with diabetes, obesity, or protein deficiency [1,2]. Cookies are considered comfort foods that may support a strict diet or a lifestyle demanding exclusion of certain nutrients (e.g., gluten) and can be subject to protein enrichment [3].

On the other hand, protein addition can have both positive and negative effects on food quality, depending on the amount and type of added protein. On the positive side, an increased nutritional value can be undoubtedly beneficial for the organism [4]. However, added protein can also contribute to the structure, consistency, and texture of food, including cookies. Certain proteins contribute to the browning and color development of cookies during baking, ensuring a more attractive appearance [5]. Nevertheless, an excessive amount of protein can result in increased hardness. Some proteins have a too strong, bitter taste, which is not desirable in cookies, thus these must be covered by extra sweetening or by using different aromas [6]. Overall, moderate protein enrichment can have a positive effect on the quality of cookies, but to overcome the downsides, it is important to choose the right type and amount of protein.

As regards protein sources, the two most common ones used to develop protein-enriched products are isolated soy protein and whey protein. Both are widely used in several ways to enhance product functionality [7]. However, whey protein is not a preferable choice for people with milk protein allergy or those following a vegan diet. The number of the latter has increased significantly in many developed countries, and it is likely that their influence on the food sector will continue to increase. Recent changes in consumer behavior also support the use of exclusively plant-based protein sources [2]. According to previous studies, emerging reasons for choosing a vegan diet are ethical, health-related, and environmental, in addition to production costs, individual taste, and religion [8,9,10,11]. Even though the increased consumption of plant-based foods is commonly considered healthy [12], concerns may arise about vegan diets regarding the absorption and availability of certain micronutrients (such as iron, iodine, vitamins A, D or B_12_, calcium, and zinc). Therefore, in order to overcome micronutrient deficiencies, a well-planned and structured vegan diet should include a variety of plants and proper fortified food, completed with sufficient sunlight exposure [13]. As regards protein intake, most literature reports have documented a gradient of protein intake among adults in Western countries, from meat eaters to vegans. Even though a vegetarian diet frequently has a reduced protein intake, it is generally still adequate in terms of the gross amount of protein. However, according to literature study findings, a smaller percentage of vegans may consume insufficient amounts of protein, but this phenomenon may be concealed by the considerably greater and ample protein intake of the general population. It is estimated that 16.5% of males and 8.1% of females within vegan eaters have a low protein consumption [14]. If a vegetarian diet excludes protein-rich foods like legumes, which are the most common source, as well as nuts, seeds, or any protein-analogs of animal foods, an inadequate intake of protein may occur [15]. In a recent study, it was even recommended that the protein Recommended Dietary Allowance (0.8 g per kg of body weight per day) should be modified to include specific recommendations for vegans, due to the lower protein quality of the majority of plant-based foods compared to animal-based foods, or at the very least to emphasize the significance of a diet with a higher protein intake to better guide and ensure nutrient adequacy in vegans. A 20% increase in the protein requirement was suggested to counter nitrogen losses. In cross-sectional studies, vegan diet adherence has also been associated with reduced muscle mass in young adults (nearly 5 kg difference compared to omnivores) [16]. These figures can even be higher for celiac and vegan people, as due to the exclusion of gluten as a protein source, gluten-free products usually have a lower protein content than conventional ones [17,18,19]. It is also well known that individual plant proteins often have lower digestibility scores than animal proteins because of their imbalanced amino acid content and decreased bioavailability. This aspect should also be considered, to ensure that daily food consumption meets nutritional standards. Even with moderately diverse sources, traditional vegetarian diets supply enough protein and amino acids; nonetheless, a tiny proportion of vegans may not consume enough [20]. A recent Danish study indeed revealed that many vegan people do not consume the recommended daily amount of protein. Moreover, it was discovered that their dietary intake was deficient in several critical amino acids, including lysine, the sulfur-containing amino acids, leucine, and valine, mainly due to the fact that only a limited number of protein sources were consumed during a day [21]. As Rojas Conzuelo and co-workers pointed out, in a diet that consists of solely low-quality protein sources, the quality of the protein may be poor. However, a vegan menu can be transformed from one with low-quality protein to one with high-quality protein after some products are replaced with high-quality protein sources [20].

In response to the challenges linked to plant-based diets, intensive research has shown that regular consumption of legumes has protective effects against obesity, type 2 diabetes, and cardiovascular disease. Studies have shown that the bioactive proteins of lentils reduce plasma levels of LDL-cholesterol, triglyceride content of the liver, and adipose tissue lipoprotein lipase activity; moreover, polyphenols of lentils could prevent angiotensin II-induced hypertension and pathological changes, including vascular remodeling and vascular fibrosis [22,23]

Lentils have been qualified as cost-effective, sustainable, and eco-friendly staple foods that are nutritious, technologically functional, protein-rich, and gluten-free, gaining a place in both celiac and vegan diets [24]. Unlike other plant-based foods, lentils can represent a suitable choice, not only due to their excellent nutritional value, but also because of their high lysine content, balanced amino acid profile, and low price [25]. The amount of essential amino acids in lentil proteins achieves FAO and WHO recommendations for adults, except methionine [26]. In addition, lentil proteins may possess positive physiological properties, having a potential beneficial impact on gut microflora modulation and hypertension. Therefore, we considered it well-justified to develop cookies that are gluten-free, rich in high-quality protein, and fiber and antioxidants, and at the same time comply with the vegan lifestyle. Due to the beneficial composition of green lentils further enriched with valuable nutrients (including indispensable amino acids), a complex and balanced composition was achieved, which is suggested to complete the diet of celiac vegan people as a nutritionally valuable commodity snack. Inulin, as a prebiotic dietary fiber, is widely accepted as a low-calorie ingredient in bakery products [27] (p. 61).

Based on the aspects explained above, i.e., consumer demand for protein-enriched products, and the restrictions and risks regarding the protein intake of vegan and celiac people, our aim was to prepare commodity snacks suitable for addressing these requirements. To achieve our goals, green lentils were chosen as nutritionally valuable, gluten-free, and protein- and fiber-rich starchy legumes. The lentil-based matrix was complemented with plant-based proteins, including brown rice, yellow pea, hemp, and pumpkin seed, in order to investigate their impact on the physicochemical, technological, and sensory properties of the original lentil cookies. The additional effect of inulin was also examined to reveal the effects of combined protein and fiber enrichment. Matcha tea powder was also added to boost antioxidant properties and modulate sensory attributes.

## 2. Materials and Methods

### 2.1. Ingredients Used for Preparation of Samples

Green lentil (*Lens culinaris* Medik.) flour was used in experiments. Whole seeds were purchased in a local market in Budapest, Hungary. Lentil flour was prepared as described in detail in our previous publication [3]. Briefly, seeds were grinded using a Grindomix GM 200 knife mill (Retsch GmbH, Haan, Germany), and the thus obtained whole meal was then sieved using a manual sieve with 500 μm openings.

Powdered sugar, glucose, salt, sodium bicarbonate, matcha tea powder (Fujian Blue Lake Foods Co., Ltd., Fuzhou, Fujian, China), and margarine were purchased in a local grocery store. The margarine had 70% fat content. It was milk- and lactose-free.

In the present experiment, four different plant proteins and a long-chain inulin with no sweetness contribution were purchased from webshops: Pumpkin seed protein concentrate (Wheyprotein.hu–Buda Family Kft.): pale green powder with a characteristic pumpkin seed odor, protein content: 57.6 ± 0.1 g/100 g. Brown rice protein (Naturize Hungary Kft.): light-beige powder with a typical odor, protein content: 75.0 ± 1.3 g/100 g. Yellow split pea protein (Wheyprotein.hu–Buda Family Kft): slightly yellowish powder with a specific odor, protein content: 76.3 ± 1.6 g/100 g. Hemp seed protein (BiOrganik Online Kft.): green powder with a distinctive odor, protein content: 42.0 ± 0.8 g/100 g. Protein contents indicated were determined as described in Section 2.7. Inulin produced from chicory (Orafti^®^FTX, Beneo, Tienen, Belgium): a slightly yellow powder, inulin content: 98 g/100 g according to producer specification.

### 2.2. Preparation of Cookie Samples

Cookies were prepared based on the AACC Approved Method 10–50D [28]. Lentil cookie enrichment with protein and fiber was performed according to the same strategies as in our previous study [29]. Instead of a quarter of the flour, protein powder was applied in order to qualify cookies as a “source of protein” [30]. A part (12.6%) of the lentil flour was replaced by inulin to reach a 6 g/100 g inulin level in the final product. The cookies thereby met the criterion of a product “high in fiber” [30]. Matcha tea powder was used for enrichment, due to its outstanding antioxidant properties. The concentration of tea powder used was established by preliminary sensory tests (data not published).

The cookie doughs were prepared by weighing and mixing the appropriate amount of each ingredient using the following recipe (Table 1). The dough was covered and left to rest in the fridge for 30 min. Subsequently, it was rolled and pressed with a rolling pin to a thickness of 6 mm, then cut into circular shapes of 50 mm diameter using a metal cookie cutter and baked in an electrically heated oven for 10 min at 200 °C. Abbreviations of prepared products are listed in Table 2.

### 2.3. Reagents and Standards Used for Chemical Determinations 

All the chemicals used during analytical procedures were of reagent grade. Neocuproine (Sigma-Aldrich, product no. N1501), gallic acid (Sigma-Aldrich, product no. G7384), and (+)-catechin hydrate (Sigma-Aldrich, product no. 22110) were purchased from Merck Life Science Kft. (Budapest, Hungary). Ethanol, methanol, glacial acetic acid, Folin–Ciocalteu’s reagent, anhydrous sodium carbonate, boric acid, and methyl red were purchased from VWR International Kft. (Debrecen, Hungary). Copper (II) chloride dihydrate, trolox (Acros Organics, product no. 218940050), aluminum chloride hexahydrate, 37% hydrochloric acid, sodium hydroxide micropearls, 96% sulfuric acid, 32% *w*/*w* sodium hydroxide solution, and bromocresol green were purchased from Reanal Laborvegyszer Kft. (Budapest, Hungary).

### 2.4. Determination of Baking Loss and Geometry

Ten circular cookie pieces were weighed before baking (w_1_) and after cooling to room temperature (w_2_). Baking loss was expressed in percentage of the average weight loss during baking and cooling: (w_1_ − w_2_)/w_1_. The diameter and thickness of each cookie were determined with the help of a measuring scale. The diameter was measured from end to end at the center point of the cookie sample. After a rotation at an angle of 90°, the cookie diameter was measured again and then the average value was recorded. The thickness of the cookie sample was determined as the length between the top and the bottom. It was measured at three points (the center and two edges), and then the average value was recorded.

### 2.5. Color Measurement

The instrument used for color measurements was a portable Chroma Meter CR-410 (Konica Minolta, Inc., Tokyo, Japan). Surface color was measured in three individual cookies per sample type. After calibrating the instrument using the reference white plate CR-A44 (Konica Minolta, Inc., Tokyo, Japan), L* [lightness: (0) black—(100) white], a* [(−) green/(+) red component], and b* [(−) blue/(+) yellow component] values in the CIE LAB color space were obtained.

CIE LAB parameters (L*, a*, and b*) were taken for further calculation of the browning index (BI), which was calculated according to the equation in [31,32]: BI = [100·(X − 0.31)]/0.172, where X = (a* + 1.75·L*)/(5.645·L* + a*−3.012·b*).

Delta E (∆E) was calculated in order to express differences in color between samples [33]. If ∆E is less than 1, the difference is normally invisible to the eye, while there is an obvious color difference if ∆E > 3.

### 2.6. Determination of Antioxidant Properties

The extraction procedure was performed in duplicate, as described in detail in our previous publication [3]. Homogenized ground materials were extracted with methanol, water, and acetic acid (75:25:0.1). Total polyphenol content (TPC) was measured using Folin–Ciocalteu reagent [34]. The results were expressed as mg of gallic acid equivalents (GAE) in 100 g of cookie sample. Total flavonoid content (TFC) was determined using the aluminum chloride colorimetric method [35]. A standard curve was prepared using catechin. Results were calculated as mg catechin equivalents (CE) per 100 g of sample. The cupric(II) ion reducing capacity (CUPRAC) method [36] was used against trolox (6-hydroxy-2,5,7,8-tetramethylchroman-2-carboxylic acid) as a standard. The antioxidant activity of the cookie extracts was expressed as mmol trolox equivalents (TE) per 100 g of cookie. Three replicates were measured for each extract and for each analytical assay (2 × 3 in total for each sample) using a Helios Alpha spectrophotometer (Thermo Spectronic, Cambridge, England).

### 2.7. Determination of Crude Protein Content

Nitrogen content was measured in triplicate with the Kjeldahl procedure, where ~1.0 g of the homogenized ground sample was digested with 15 mL of concentrated sulfuric acid containing catalyst (Kjeltabs Se/3.5) using a Labtec^TM^ DT208 Digestor (FOSS Analytical Co., Ltd., Suzhou, China) unit. The resultant solution was distilled with 50 mL of NaOH using a Kjeltec^TM^ 8200 equipment (FOSS Analytical Co., Ltd., Suzhou, China). Liberated ammonia was trapped in 30 mL of 1% boric acid solution and titrations were performed with standardized 0.1N HCl to a mixed indicator endpoint (0.1 g/100 mL bromocresol green and 0.1 g/100 mL methyl red in methanol). The protein conversion factor was 6.25 for all the cookies, except for those containing brown rice protein, where 5.95 was used as a conversion factor [37].

### 2.8. pH Measurement

pH values were measured in triplicates from water extracts prepared from 5.00 g sample suspended in 10 mL water using a Testo 206-pH2 digital pH meter (Testo SE & Co. KGaA, Lenzkirch, Germany) device.

### 2.9. Texture Profile Analysis

Measurements were performed using a Brookfield CT3 Texture Analyzer (Ametek Brookfield, Middleborough, MA, USA). Sample penetration and data acquisition were controlled using the TexturePro CT v1.9 build 35 software (Ametek Brookfield, Middleborough, MA, USA) provided with the apparatus. A two-cycle program was used to allow the probe to travel 5.0 mm into the sample, return, and repeat. A TA9 probe (stainless steel needle) was used at 1 mm/s speed. Four cookies were selected, each was tested in four replicates in the central position.

The textural parameters are automatically calculated from the force–time curve by the software as follows. Hardness [g] is given as the positive peak force during the first cycle. Adhesive force [g] is defined as the negative peak measured on the first probe reversal. Cohesiveness [−] represents the ratio of positive force during the second to that during the first cycle. Gumminess [g] is calculated as hardness × cohesiveness.

### 2.10. Sensory Tests

Sensory analyses were performed on freshly baked cookies in two experiments. In the first, cookies without dietary fiber were tested with 18 participants. In the second, cookies with dietary fiber addition were evaluated by 23 panelists. A simplified profile analysis was applied, as described previously by the authors [29]. The following sensory attributes were evaluated on a 1–10 structured linear scale: surface homogeneity (cracked-homogeneous, smooth); surface color (green-brown); tea odor, baked odor, lentil taste, tea taste, and sweet taste (uncharacteristic, not perceptible-intense); hardness (soft-hard); crunchiness (sticky, chewy-crunchy); crumbliness (crumbly, dry-not crumbly).

### 2.11. Data Analysis

In the case of texture parameters, data screening was applied to remove outliers, as in our previous papers [3,29]. Among the sixteen measured data, the two lowest and the two highest data were skipped. Statistical analyses were carried out using Statistica ver.13.5.0.17. (TIBCO Software Inc., Palo Alto, CA, USA) software at a significance level (α) of 0.05: one-sample Student’s *t*-test for comparing different sample types with the control cookie (only in the case of sensory data) and Tukey’s post hoc test for pairwise comparisons. Principal components analysis (PCA) was also used for finding patterns in the datasets.

## 3. Results and Discussion

### 3.1. Physicochemical Properties of Vegan Lentil Cookies

#### 3.1.1. Color

**Figure 1:**
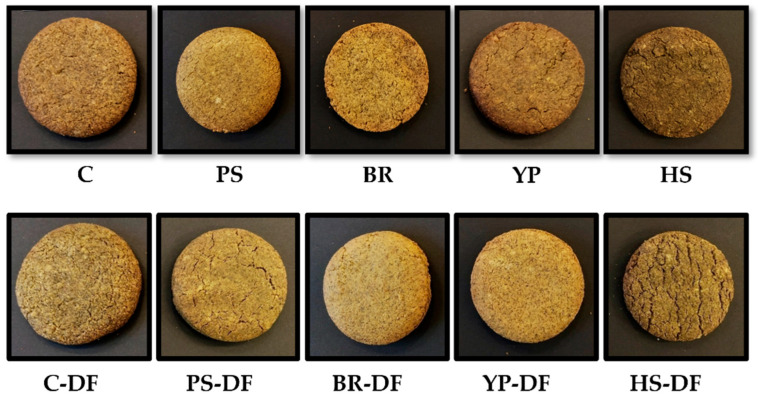
Green lentil cookies prepared using different plant proteins and/or inulin. (C: control, DF: dietary fiber, BR: brown rice, HS: hemp seed, PS: pumpkin seed, YP: yellow split pea. The composition of the cookie samples is shown in Table 2).

The impact of protein enrichment on the color of the cookies (Table 3, Figure 1) did not show a clear trend: depending on the type of protein, the samples became lighter (BR, YP), darker (PS, HS), less reddish (BR), more yellowish (BR, YP), or less yellowish (HS). All the proteins impacted at least one of the color values measured, while the largest color difference was observed in the case of brown rice and split pea protein addition. The latter produced the largest color difference for both inulin-free and inulin-enriched samples, despite of the fact that the original color of this protein was not an intensive one. However, as other authors also noticed, the natural color of the proteins had an effect on the color parameters of the product [38]. This color lightening and yellowishness can be partly attributed to the pale color of the yellow pea and brown rice proteins, while darkening of the cookies and their shift towards blue is partly explained by the color of hemp seed protein; however, this was not the case for the pale green pumpkin seed protein. A possible explanation for the latter may be the presence of both carotenoid and chlorophyll residues in pumpkin seed and only chlorophyll residues in hemp seed proteins [4].

Addition of fiber had a smaller impact on color, especially in the case of brown rice and yellow pea proteins, as well as the control, where no significant changes occurred. For both pumpkin seed and hemp seed protein cookies, inulin addition resulted in lighter, less reddish and more yellowish products, which is not necessarily a positive change from the point of view of consumer acceptance. Color differences compared to the inulin-containing control were generally smaller in the presence of fiber.

Color development during baking is a function of the ingredients, pH, and the baking conditions (temperature, time, and air velocity in the oven) [39,40]. Moreover, it is not simply the chemical transformations of the individual components (i.e., mainly the Maillard and caramelization processes), but also the bonds established between them. As uniform conditions were ensured for all baking lots, the color differences can clearly be attributed to the difference in the composition of the proteins used and, to a lesser extent, to the presence of the inulin. As the Maillard reaction advances, amino groups are consumed, sugars are degraded, and peptides and free amino acids can contribute to the development of acidic compounds [41]. This is one of the possible explanations for the substantial color changes as functions of the protein type added. In addition to the covalent bonds formed in the Maillard reaction or other chemical cross-linking reactions, proteins and sugars are also known to interact by forming non-covalent bonds, these include electrostatic interaction, hydrogen bonds, hydrophobic interaction, and van der Waals forces, as demonstrated in the case of whey protein [42]. Moreover, protein conformation, as well as the chemical and steric characteristics of the protein surface have also been reported to impact protein’s ability to interact with other proteins, polysaccharides, or lipids, which is crucial for protein techno-functionality [43]. Finally, polyphenols may also play a role in color formation by suppressing the Maillard reaction, as was confirmed for epicatechins from green tea, which were also present in our products [44]. Polyphenols can also form conjugate complexes with proteins [45]. Thus, the different amino acid profile of the proteins added into the composition resulted in different reaction rates, leading to different amounts of products formed in the reactions above. It is widely acknowledged that the carbonyl group of reducing sugars combines with the amino group of a protein—free lysine being the most reactive—during the initial stage of the Maillard reaction. The carbonyl groups of reducing carbohydrates and the ε-amino groups of lysine or, to a lesser extent, the α-amino groups of terminal amino acids and the imidazole and indole groups of histidine and tryptophan are the first reaction of the first stage. Nevertheless, most of the color is formed in the reaction’s final stage, when melanoidins are generated. Apart from the lysine ε-amino groups, protein-bound arginine can also react in the later stages of the process, with its guanidino groups [42,43]. Polyphenol oxidation compounds, on the other hand, can bind spontaneously to the nucleophilic moieties of amino acids and proteins, e.g., the ε-amino group of lysine or the sulfhydryl group of cysteine, forming stable, covalent bonds and thus having an impact on the overall outcome of the heat treatment [46]. Pea protein isolate was reported to be rich in lysine (4.7 g/100 g [47]), thus free lysine groups could account for the high browning index observed for the pea protein-enriched cookies. Brown rice, having a lower lysine content (1.9 g/100 g [47]), showed a similar browning index value in our cookies enriched with this protein. Pumpkin seed has an even lower lysine content (1.7 g/100 g) [48]; and hemp seed has the lowest lysine level (1.4 g/100 g) [47]. These lysine levels were not reflected in the browning indices obtained for the yellow pea and brown rice protein-enriched cookies, presumably due—among other possible factors—to the colors of the proteins themselves. Finally, it should be noted that pH, as a major factor impacting the Maillard reaction, was not dramatically shifted by the addition of proteins: it was 7.04 for the control, and it ranged from 6.78 to 7.10 for the protein-enriched cookies. Inulin addition produced only minor changes in pH (7.00 for the fiber-enriched control, values ranging from 6.75 to 7.31 for the protein- and fiber-enriched samples).

The color differences found for the pumpkin seed, yellow pea, and hemp seed protein cookies were overall lower than those measured by Nemś et al. [4] in wheat-based enriched cookies, which is attributed to the color of the green lentil flour used. In accordance with the same authors [4], the hemp seed protein-enriched samples were the darkest of all.

#### 3.1.2. Total Polyphenol and Flavonoid Content, Antioxidant Capacity, and Crude Protein Content

Protein addition resulted generally in a change in the total polyphenol content of the samples, whether enriched or not with inulin (Table 4). All the protein-enriched cookies had lower polyphenol content than the controls. Among all the proteins used, hemp seed protein produced the highest TPC value, which is in agreement with other authors’ observations, and was probably connected to the endogenous polyphenol content of the hemp seed product commercialized with a relatively low protein content [49]. The total flavonoid content was not impacted significantly in all the protein-added cookies: for the inulin-free samples, pumpkin seed and yellow pea proteins did not produce statistically relevant changes, while for the inulin-enriched samples, hemp seed protein did not induce relevant modifications in the flavonoid content.. Interestingly, brown rice protein produced high values in both groups, but hemp seed protein only boosted flavonoid content in inulin-free samples. In terms of copper(II) ion reducing activity, protein addition resulted in an overall decrease in the antioxidant capacity. When all samples are considered together, the values were overall higher for the inulin-free samples.

Crude protein content was significantly boosted upon protein enrichment, with only one exception (hemp seed protein + inulin addition), attributed to the lower initial protein content of the hemp seed protein product used. The highest values were obtained in both groups for brown rice and yellow pea proteins, in accordance with the purity of the products used. It was noteworthy that, due to the protein enrichment applied, all the cookies qualified as “sources of protein” according to the official nutrition claims of the European Union stated in Regulation EC) No 1924/2006 [30].

#### 3.1.3. Baking Loss and Geometry

Baking loss (Table 5) only significantly increased in the yellow pea protein-containing cookies, remaining unchanged in the rest. The results are in line with those obtained for whey protein-enriched lentil cookies, which did not cause a change in baking loss [29]. According to Sahagún et al., water holding and water binding capacities are strongly related to the type of the protein [5], which may provide an explanation for the results obtained with different proteins (explained in detail below).

The addition of proteins in all cases resulted in a decrease in the diameter, with yellow pea protein producing the smallest diameter, in accordance with the highest baking loss observed for this protein. The height remained unchanged in the presence of pumpkin seed protein and increased significantly for the hemp seed and brown rice proteins, while it decreased for the yellow pea sample (the latter is again explained by the high baking loss).

Spread ratio (showing the quality of the flour used and the ability of the cookie to rise [50]) is generally considered to be preferably higher for cookies [39]. A greater spread is specific to dough ingredients with poor hydration qualities and poor water retention capacity [51]. Our results only showed an increase for yellow pea-containing cookies, for all the other samples, the spread ratio decreased compared to the control. This may be explained by the water holding capacity of yellow pea proteins. It was shown that pea protein isolate in its natural form has a high water holding capacity, on its own [4,52] and in batters [53]. The water holding capacity of untreated pea protein is not lower compared to rice or hemp seed proteins; however, at basic pH and upon heat treatment (conditions that are similar to our case), this property undergoes a dramatic change and suffers a substantial decrease [52]. Nevertheless, pea protein isolate was also shown to have a relative high solubility, which may also have contributed to the results obtained, as solubility is negatively correlated to water holding capacity [52,54]. On the other hand, hemp and pumpkin seed proteins have similar water holding capacities [4], which was also reflected in the results obtained by us. A decrease in the spread ratio relative to the control was also observed for the inulin-enriched samples, including the yellow pea-containing samples. The inulin probably compensated for the latter effect produced on spread ratio when yellow pea protein was used alone.

It was reported by Drakos et al. that elevated of levels of inulin (20–30%) led to a decrease in the spread ratio of wheat–barley cookies, but there were no differences in spread ratio at 10% inulin enrichment. They suggested that an increase in fiber content can lead to a decreased spread ratio; however, this also depends on the fiber type, and components with different water absorption capacities had lower spread ratios. These factors may be connected to the formation of aggregates in composite flours, with more hydrophilic sites available to compete for the limited free water in the dough. Spread ratio is also connected to the protein and the damaged starch content of flours. Protein molecules can provide hydrophilic sites that also compete for the free water. Rapid partitioning of free water by these hydrophilic sites results in higher sugar concentrations in the water phase, higher internal dough viscosity, and thus, limited cookie spread [55]. It is thus understandable that different plant-based proteins will form different hydrophilic sites, resulting in various spread ratios in the cookies. The particular protein composition and structure of each protein, their water absorption capacity, and their interactions with the fiber and starch content of the dough may possibly be behind the results obtained.

Inulin addition only led to an increase in the baking loss for the control, while the other parameters remained unchanged for this sample compared to the inulin-free one. The increase in baking loss as a result of fiber addition is in line with results observed by us previously [29] and is also in accordance with other reports [29,56]. The higher baking loss is attributed to the lower water retention capacity of inulin in comparison to the green lentil flour, which was partly replaced in the recipe by inulin. As regards the protein-enriched cookies, the baking loss was only significantly different from the control in the case of the pumpkin seed protein-containing cookie, which is a very different situation compared to the baking losses of the samples containing no inulin, where only yellow pea protein produced a significantly different baking loss. When the samples from the two groups (inulin-enriched or not) were compared, the baking loss differences proved not to be significantly impacted upon inulin addition (similarly to observed for the whey-inulin combination [29]). On the other hand, both diameter and height increased, together with a subsequent decrease in the spread ratio for yellow pea-containing cookies. The other sample that underwent significant changes in its geometry was the hemp seed protein-containing sample, where a decrease in height and a concomitant increase in the spread ratio were observed upon fiber enrichment. Apart from these two samples, inulin addition did not result in significant changes in geometry and spread ratios compared to the corresponding inulin-free samples. This phenomenon was similar in the case of whey protein enrichment [29]. By adding red kidney bean protein isolate to wheat cookies at 0–20%, Hayat et al. observed a gradual decrease in the diameter and spread ratio, together with a height increase [57], which appears to be different from the results observed by us for yellow pea protein.

### 3.2. Texture Properties of Vegan Lentil Cookies

Among the protein-enriched compositions examined (Table 6), samples containing yellow pea, hemp seed, and brown rice proteins proved to show the highest variability in terms of texture properties, especially hardness and cohesiveness. Hardness is one of the basic features for assessing the texture of shortcrust cookies [4]. Compared to the unenriched control, samples containing brown rice protein were significantly softer and showed higher cohesiveness, i.e., they were stickier. On the other hand, samples containing split pea protein were harder, less sticky (lower cohesiveness) and less gummy than the control. Finally, cookies prepared with hemp seed protein were stickier and gummier than the control cookies. Pumpkin seed protein addition produced the most similar texture in comparison with the control.

Fiber addition generally resulted in a significant decrease in hardness, especially in the case of yellow pea protein; however, hemp seed protein-containing cookies became harder in the presence of inulin. On the other hand, cohesiveness increased in most of the inulin-enriched samples, with hemp seed proteins again producing an inverse effect in terms of this parameter. A lower cohesiveness upon pea protein addition was also reported in enriched focaccia products [58]. Adhesive force did not show major changes upon fiber enrichment, but gumminess was reduced in most cases, with hemp seed protein as an exception in all cases.

Research over the last thirty years has demonstrated that many of the techno-functional qualities of dietary proteins are dramatically changed by glycation with sugars through the Maillard reaction when heated [43,59,60]. Not only smaller carbohydrate molecules, but also polysaccharides interact with proteins, modifying their secondary or tertiary structures and thus impacting texture properties through protein cross-linking formation, emulsion properties, and protein–polysaccharides conjugates formation [44]. Moreover, in a ternary system containing proteins, polysaccharides, and polyphenols (which is the case of our cookies), these can interact in a way (hydrogen bond, hydrophobic, and electrostatic interactions) that has further impacts on the functional properties of the product [61]. Thus, complex processes are assumed to take part during baking that strongly depend, not only on the individual behavior exerted by the plant proteins used, but also on their interactions with the other components under the pH, temperature, and time conditions of the thermal treatment.

An increase in hardness upon pea protein addition was previously observed by other authors, including De Angelis et al. [58]. Nems et al. used pea, hemp, and pumpkin seed proteins to enrich wheat cookies and found that 10% pea and pumpkin seed protein addition resulted in increased hardness, while hemp seed protein provided a softer texture compared to the control cookies. They attributed the differences obtained to the different dietary fiber content of the protein preparations used, which may be a further factor contributing to the texture differences observed (higher endogenous fiber content resulted in softer cookies) [4]. This inverse relationship between fiber content and hardness was also confirmed for some soluble fibers, including fructoligosaccharides [62,63], but the opposite was noticed for cookies enriched with a soybean-derived by-product rich in soluble fiber [64]. In our case, the matrix used was substantially different, in terms of the high fiber content of the green lentil used (30 g/100 g according to [65]), so the addition the proteins decreased the original fiber content of the cookies. This can provide an explanation for the results observed: hardness increased upon adding pumpkin seed protein (with the second highest fiber content, 13.2 g/100 g), even though it did not produce a statistically significant increase in hardness. Hemp seed protein-enriched cookies were not softer than the controls in any of the groups (with or without inulin), despite the high fiber content of the protein used (21 g/100 g). Yellow pea protein produced a dramatic change in hardness, which can be partly explained by its low fiber content (0.5 g/100 g); however, brown rice protein with a similarly low fiber content (3.6 g/100 g) led to the softest cookies of all. It thus seems that fiber content is one, but not always the main factor in determining changes in the hardness of cookies. Nevertheless, the overall hardness-decreasing effect of increased fiber content was confirmed for the inulin-enriched cookies.

### 3.3. Sensory Properties of Vegan Lentil Cookies

The results of the sensory panel test (Table 7) show that in samples containing no added fiber the surface homogeneity was significantly worse in the protein-enriched cookies compared to the control. However, this effect was compensated by inulin addition, which resulted in more homogeneous surfaces for the brown rice and yellow pea protein-containing samples, but the surface homogeneity worsened for inulin-enriched hemp seed protein-containing cookies.

As regards the green color of the cookies, produced mainly by the matcha tea component and the inherent color of some of the proteins, this was perceived as more intensive in the hemp seed protein-containing cookies; however, upon inulin enrichment, the intensity of the green color decreased for this sample and increased for those containing pumpkin seed, brown rice, and yellow pea proteins. The predominant color difference obtained upon hemp seed protein enrichment is in line with the observations of Nems et al. [4], who also found smaller differences for cookies containing pumpkin seed and pea proteins.

Tea odor was negatively impacted by addition of all proteins; and the results were not impacted by inulin addition. On the contrary, baked odor was generally enhanced upon protein addition, the only exception here being the yellow pea protein, which produced no significant change in comparison with the unenriched control.

The Maillard process during baking produces a variety of volatile chemicals. Higher protein levels may influence the Maillard reaction rate indirectly through hydrolysis or deamination of bound amino acids. Higher protein content during baking encourages the production of pyrazines, which give food a roasted flavor sensation [57]. This is in line with the significantly higher baked odor values observed for all the protein-enriched cookies, the only exception being (as for many other measured parameters) the yellow pea protein, which did not differ significantly from the controls.

Tea taste was also negatively impacted by protein addition, the only exceptions being hemp seed protein and yellow pea protein, the latter in combination with inulin. On the other hand, sweet taste was not influenced by either protein or protein + inulin addition, the only exception here being the yellow pea protein, whether combined with inulin (Table 8). The lentil taste was apparently not masked by any protein addition; however, it was intensified when yellow pea protein and inulin were added together. The legume notes seem to be a dominant factor in determining the overall sensory properties of bakery products. In focaccia samples containing rice, corn flour, and pea protein concentrate, the perception of the “legume odor” was noted for all the compositions, having become negligible only at a level as low as 2 g/100 g of pea protein concentrate [58]. Polat et al. also observed that the addition of 15% germinated green lentils significantly affected the color, taste, and flavor of wheat-based crackers [66].

Only some of the proteins had a significant effect on hardness and crunchiness: in the absence of inulin, the yellow pea protein-containing cookies were perceived as harder and crunchier, while hemp seed protein-containing ones were softer and less crunchy than the control. Interestingly, this trend changed upon inulin addition: the first ones became softer and less crunchy, the second ones harder and crunchier than the corresponding control. These results are in line with those from the instrumental hardness measurements. In the absence of inulin, the cookies became crumblier upon protein enrichment (again, yellow pea protein was an exception to this trend). When inulin was added to the dough, all cookies were crumblier, except for the hemp seed protein-enriched ones.

### 3.4. Principal Components Analysis

Principal components analysis (PCA) was performed to check for possible differentiation patterns between samples, based on the attributes measured.

**Figure 2:**
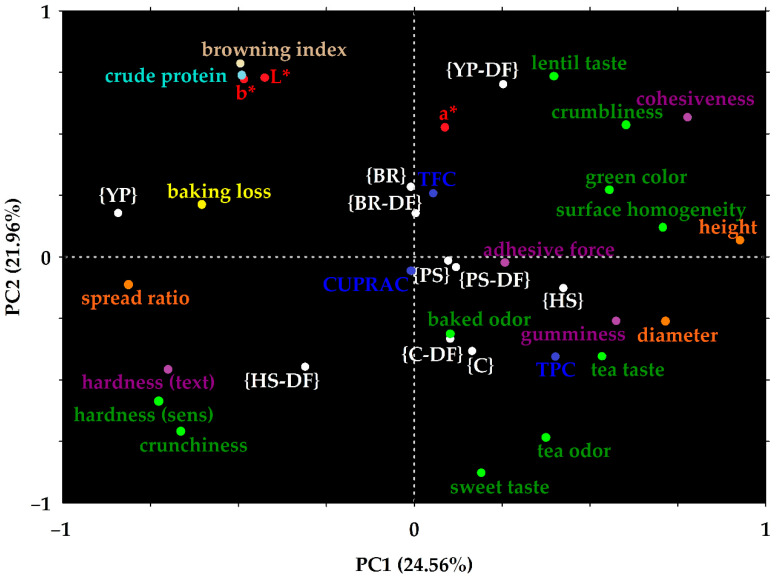
Principal components analysis (PCA) of the dataset. Each colored dot represents an attribute studied (green: sensory properties, orange: geometry, blue: antioxidants, light blue: crude protein, yellow: baking loss, brown: browning index, red: color parameters, purple: texture properties). Hardness was measured by sensory (sens) and instrumental (instr) methods (CUPRAC: cupric ion reducing antioxidant capacity, TFC: total flavonoid content, TPC: total polyphenol content). White dots represent the different samples. (C: control, DF: dietary fiber, BR: brown rice, HS: hemp seed, PS: pumpkin seed, YP: yellow split pea. The composition of the cookie samples is shown in Table 2.)

Four principal components were calculated that covered 72.76% of total variance (see Tables S1 and S2). However, the first two principal components (PC1 and PC2) covered 46.52% of the total variance. Their plot (Figure 2) showed that results of sensorial and instrumental measurement methods for the same parameters presented similar tendencies, as indicated by their close proximity (for example crumbliness and cohesiveness, green color and a*). Tea notes seemed to be linked not only to the total polyphenol content, but also to sweet taste and negatively linked to lentil taste, which supports the use of matcha tea as a flavor corrector.

Each type of plant protein added to the lentil cookies changed the characteristic of the control. The biggest difference was observed in the case of yellow pea protein, which was distinguished from the rest of the samples in terms of baking properties (baking loss, spread ratio).

Hemp seed protein and inulin together increased hardness and crunchiness. Pumpkin seed and brown rice proteins had a positive impact on antioxidant properties, the first protein and hemp seed protein (without added fiber) being the closest to the unenriched control.

## 4. Conclusions

Plant-based protein preparations (pumpkin seed, brown rice, yellow pea, and hemp seed) can be used to replace part of the lentil flour in green lentil cookies, thus increasing their protein ratio and shaping their physicochemical and functional, as well as sensory properties, in various ways. The impact of inulin, as a prebiotic fiber, was also tested in all protein-enriched combinations, and matcha tea powder was added as a nutritionally beneficial component.

The protein contents of the enriched cookies were boosted, reaching up to 20% in the case of pure isolates. However, as expected, total polyphenol content and antioxidant capacity were generally lower upon replacement of a quarter part of lentil flour with proteins and further reduced when fiber was added.

In most cases, the color of the cookies changed in a visibly perceivable way. This color modification was attributed, on the one hand, to the endogenous color of the protein preparations, on the other hand, to the impact of the proteins on multiple processes taking place during baking. This latter fact applied especially to yellow pea protein, which produced lighter cookies but high browning indices. The baking loss and geometry values obtained showed that both proteins and inulin produced significant changes. Again, yellow pea protein-enriched cookies produced extreme values, which were moderated by the presence of inulin.

The texture properties proved to be highly impacted in samples containing yellow pea, hemp seed, and brown rice proteins, especially in terms of hardness and cohesiveness. For the inulin-enriched cookies, the general hardness-decreasing effect of the fiber content was confirmed, especially in the case of yellow pea protein.

The sensory panel test demonstrated that the surface homogeneity of the protein-enriched cookies was considerably lower in samples without additional fiber than in the control. However, the inulin offset this effect for many samples. As regards the green color of the protein-enriched cookies, excepting the hemp seed-containing cookies, this was not different from the controls. Hardness and crumbliness were perceived as in line with the results of the instrumental measurements. The crunchiness of yellow pea and hemp seed protein-enriched cookies was substantially affected by inulin addition. Tea notes were partially masked by all proteins, while inulin did not show such an effect. As a result of the enhanced Maillard process, the baked odor was more prominent for all protein-containing products, while sweet taste was only perceived to be weaker in the yellow pea cookies.

Based on our findings, it may be concluded that plant-based protein enrichment increases the nutritional quality of lentil cookies in terms of enhanced crude protein content; however, the amount of supplementation is primarily limited by the color and taste of the products. Further studies are envisaged to find the ideal combination of plant proteins to ensure a nutritionally complete amino acid profile, as well as suitable texture, color, and sensory characteristics.

## Figures and Tables

**Table 1:** Recipe of vegan cookie samples.

Ingredient	Control	With Added Protein	With Added Fiber and Protein
Green lentil flour	100.0 g	75.0 g	62.4 g
Powdered sugar	57.8 g	57.8 g	57.8 g
Margarine	28.4 g	28.4 g	28.4 g
Salt	0.93 g	0.93 g	0.93 g
Sodium bicarbonate	1.11 g	1.11 g	1.11 g
Distilled water	7.11 g	7.11 g	7.11 g
Glucose solution (5 g/ 100 mL)	14.6 g	14.6 g	14.6 g
Protein powder	--	25.0 g	25.0 g
Inulin	--	--	12.6 g
Matcha tea	4.2 g	4.2 g	4.2 g

**Table 2:** Abbreviations used for vegan lentil cookies.

Protein Type	Without Dietary Fiber	With Dietary Fiber
No added protein	C	C-DF
Pumpkin seed protein	PS	PS-DF
Brown rice protein	BR	BR-DF
Yellow split pea protein	YP	YP-DF
Hemp seed protein	HS	HS-DF

**Table 3:** Color parameters of vegan lentil cookies.

Sample ^1^	L*	a*	b*	BI ^2^	ΔE* ^3^	
C	36.45 ± 0.84 C,bc	2.22 ± 0.18 B,ab	13.98 ± 0.38 B,bc	51.11 ± 0.54 B,b	--	--
PS	34.52 ± 0.48 B,ab	2.48 ± 0.43 B,bc	13.11 ± 0.33 B,b	51.39 ± 1.37 B,b	2.13	--
BR	42.32 ± 0.17 D,d	1.19 ± 0.11 A,a	20.11 ± 0.09 C,d	63.45 ± 0.55 C,cd	8.55	--
YP	42.71 ± 0.71 D,d	2.74 ± 0.36 B,bc	20.30 ± 0.31 C,d	66.36 ± 1.04 C,d	8.92	--
HS	32.29 ± 0.72 A,a	2.47 ± 0.46 B,bc	10.25 ± 0.55 A,a	42.63 ± 2.15 A,a	5.59	--
C-DF	36.72 ± 0.81 A,bc	1.03 ± −0.74 A,a	14.29 ± 0.09 A,c	49.49 ± 2.67 A,b	--	1.26
PS-DF	36.94 ± 0.62 A,c	1.77 ± 0.40 A,ab	14.57 ± 0.66 A,c	51.80 ± 0.88 A,b	0.84	2.92
BR-DF	42.49 ± 0.23 B,d	1.05 ± 0.07 A,a	19.42 ± 0.15 B,d	60.13 ± 0.55 B,c	5.80	0.72
YP-DF	42.22 ± 1.56 B,d	3.64 ± 0.75 B,c	19.44 ± 0.45 B,d	65.58 ± 2.99 C,d	7.97	1.34
HS-DF	35.00 ± 0.96 A,bc	1.04 ± 0.16 A,a	13.72 ± 0.39 A,bc	50.03 ± 0.55 A,b	1.82	4.62

Data presented as mean ± standard deviation (*n* = 3). Within each column different capital letters and lowercase letters represent significant differences between means according to Tukey’s HSD test (one-way ANOVA) at a α = 0.05 confidence level. Capital letters indicate statistical differences within the two groups (samples without inulin and samples enriched with inulin), while lowercase letters indicate statistical differences within all samples. ^1^ The composition and abbreviations of the cookie samples are given in Table 2. ^2^ BI, browning index. ^3^ ΔE* values in left column represent the color difference compared to the color of the control cookie from the same group. The values in the right column show the color differences between samples without inulin and samples enriched with inulin.

**Table 4:** Total polyphenol (TPC) and total flavonoid (TFC) content, cupric ion reducing antioxidant capacity (CUPRAC), and crude protein (CP) content of vegan lentil cookies.

Sample ^1^	TPC mg GAE/100 g	TFC mg CE/100 g	CUPRAC mmol TE/100 g	CP ^2^ g/100 g
C	552 ± 13 D,h	133 ± 9 AB,cd	3.13 ± 0.07 D,f	12.4 ± 0.8 A,a
PS	410 ± 12 B,ef	128 ± 11 A,c	2.14 ± 0.05 A,c	17.1 ± 0.7 C,d
BR	391 ± 9 B,de	186 ± 10 C,e	2.15 ± 0.06 A,c	19.0 ± 0.2 D,ef
YP	356 ± 21 A,c	152 ± 11 B,d	2.69 ± 0.05 C,e	20.4 ± 0.2 E,f
HS	463 ± 16 C,g	208 ± 5 C,e	2.39 ± 0.08 B,d	15.1 ± 0.3 B,bc
C-DF	425 ± 11 D,f	80.1 ± 6.3 B,b	2.17 ± 0.06 C,c	12.5 ± 0.1 A,a
PS-DF	368 ± 13 C,cd	36.2 ± 6.6 A,a	1.92 ± 0.05 B,b	15.9 ± 1.0 BC,cd
BR-DF	261 ± 6 A,a	125 ± 2 C,c	1.47 ± 0.09 A,a	16.5 ± 1.2 C,cd
YP-DF	349 ± 12 C,c	122 ± 3 C,c	2.27 ± 0.17 C,cd	17.2 ± 0.4 C,de
HS-DF	311 ± 6 B,b	89.1 ± 16.9 B,b	1.58 ± 0.01 A,a	14.0 ± 0.1 AB,ab

Data presented as mean ± standard deviation (*n* = 5 for antioxidant measurements and *n* = 3 for crude protein determination). Within each column different capital letters and lowercase letters represent significant differences between means according to Tukey’s HSD test (one-way ANOVA) at a α = 0.05 confidence level. Capital letters indicate statistical differences within the two groups (samples without inulin and samples enriched with inulin), while lowercase letters indicate statistical differences within the group including all the samples. ^1^ The composition and abbreviations of the cookie samples are given in Table 2. GAE: gallic acid equivalent, TE: trolox equivalent, CE: catechin equivalent. ^2^ The nitrogen-to-protein conversion factor was 5.95 for samples containing brown rice protein. The factor 6.25 was used for the calculation of crude protein content in the other cases.

**Table 5:** Baking loss and geometry of vegan lentil cookies.

Sample ^1^	Baking Loss, %	Diameter, mm	Height, mm	Spread Ratio (Diameter/Height)
C	12.6 ± 4.5 A,a	56.8 ± 1.2 D,f	8.5 ± 0.3 B,cd	6.69 ± 0.30 C,f
PS	13.8 ± 0.9 A,abc	53.0 ± 1.0 C,d	8.3 ± 0.3 B,bc	6.39 ± 0.24 B,de
BR	12.3 ± 1.1 A,a	50.8 ± 0.6 B,b	8.9 ± 0.1 C,e	5.71 ± 0.09 A,ab
YP	17.9 ± 2.2 B,d	47.8 ± 0.5 A,a	5.7 ± 0.1 A,a	8.38 ± 0.19 D,g
HS	13.3 ± 0.9 A,ab	52.4 ± 1.1 C,cd	9.3 ± 0.1 D,f	5.63 ± 0.11 A,a
C-DF	16.1 ± 1.9 C,cd	56.8 ± 0.6 D,f	8.6 ± 0.2 B,d	6.60 ± 0.16 C,ef
PS-DF	12.4 ± 1.3 A,a	53.3 ± 0.8 B,d	8.6 ± 0.2 B,d	6.20 ± 0.20 B,cd
BR-DF	14.4 ± 1.1 B,abc	52.1 ± 0.8 A,bcd	8.9 ± 0.1 C,e	5.85 ± 0.11 A,ab
YP-DF	15.7 ± 0.8 BC,bcd	54.8 ± 1.2 C,e	9.2 ± 0.2 D,f	5.96 ± 0.23 A,bc
HS-DF	14.5 ± 0.8 B,abc	51.2 ± 0.9 A,bc	8.1 ± 0.1 A,b	6.32 ± 0.16 B,d

Data presented as mean ± standard deviation (*n* = 10). Within each column different capital letters and lowercase letters represent significant differences between means according to Tukey’s HSD test (one-way ANOVA) at a α = 0.05 confidence level. Capital letters indicate statistical differences within the two groups (samples without inulin and samples enriched with inulin), while lowercase letters indicate statistical differences within all samples. ^1^ The composition and abbreviations of the cookie samples are given in Table 2.

**Table 6:** Texture properties of vegan lentil cookies.

Sample ^1^	Hardness, g	Adhesive Force, g	Cohesiveness, -	Gumminess, g
C	817 ± 186 B,d	106 ± 30.1 AB,ab	0.092 ± 0.012 B,b	74.6 ± 23.2 B,cd
PS	926 ± 114 B,d	110 ± 84.5 AB,ab	0.098 ± 0.012 B,bc	91.2 ± 17.6 BC,de
BR	629 ± 88 A,c	170 ± 3.6 B,b	0.126 ± 0.008 C,d	81.8 ± 3.6 BC,cde
YP	1354 ± 216 C,f	72.7 ± 62.9 A,a	0.030 ± 0.018 A,a	39.0 ± 18.3 A,a
HS	820 ± 94 B,d	124 ± 76.9 AB,ab	0.118 ± 0.014 C,d	98.1 ± 20.5 C,e
C-DF	557 ± 104 C,bc	81.8 ± 31.8 A,a	0.115 ± 0.012 B,cd	63.7 ± 12.0 C,bc
PS-DF	426 ± 47 B,b	94.8 ± 38.5 A,a	0.127 ± 0.012 B,d	55.1 ± 8.4 BC,ab
BR-DF	473 ± 64 BC,bc	105 ± 20.2 A,ab	0.120 ± 0.009 B,d	55.2 ± 9.6 BC,ab
YP-DF	213 ± 22 A,a	84.9 ± 18.6 A,a	0.184 ± 0.018 C,e	40.2 ± 5.7 A,a
HS-DF	1190 ± 123 D,e	118 ± 97.4 A,ab	0.036 ± 0.008 A,a	44.9 ± 12.0 AB,ab

Data presented as mean ± standard deviation (*n* = 12). Within each column, different capital letters and lowercase letters represent significant differences between means according to Tukey’s HSD test (one-way ANOVA) at a α = 0.05 confidence level. Capital letters indicate statistical differences within the two groups (samples without inulin and samples enriched with inulin), while lowercase letters indicate statistical differences within all samples. ^1^ The composition and abbreviations of the cookie samples are given in Table 2.

**Table 7:** Sensory properties of vegan lentil cookies.

Sample ^1^	Surface Homogeneity	Green Color	Tea Odor	Baked Odor	Tea Taste
C	7.0 A	4.0 A	8.0 A	3.0 A	7.0 A
PS	6.2 ± 1.6 B,b	4.6 ± 2.0 A,ab	5.1 ± 3.4 B,a	4.9 ± 2.2 B,ab	4.7 ± 2.5 B,a
BR	5.1 ± 1.9 B,b	3.3 ± 2.3 A,a	4.5 ± 3.1 B,a	4.7 ± 2.5 B,ab	3.6 ± 2.8 B,a
YP	2.9 ± 1.2 B,a	3.6 ± 2.4 A,a	3.4 ± 2.8 B,a	2.9 ± 2.4 A,a	4.6 ± 2.9 B,a
HS	5.2 ± 1.8 B,b	6.4 ± 2.3 B,b	5.2 ± 3.8 B,a	5.4 ± 2.5 B,b	7.0 ± 2.1 A,b
C-DF	7.0 A	4.0 A	8.0 A	3.0 A	7.0 A
PS-DF	6.0 ± 2.2 B,b	5.0 ± 2.2 B,a	5.0 ± 2.6 B,a	4.9 ± 2.5 B,b	6.0 ± 2.1 B,a
BR-DF	7.1 ± 2.4 A,b	6.0 ± 2.8 B,a	4.2 ± 2.7 B,a	4.7 ± 2.5 B,b	5.4 ± 2.4 B,a
YP-DF	6.3 ± 2.5 A,b	6.1 ± 3.1 B,a	3.8 ± 2.8 B,a	2.8 ± 2.7 A,a	6.3 ± 2.9 A,a
HS-DF	2.1 ± 1.7 B,a	4.6 ± 3.1 A,a	5.2 ± 3.1 B,a	6.2 ± 2.4 B,b	5.6 ± 2.4 B,a

Data presented as mean ± standard deviation. Within each column different capital letters and lowercase letters represent significant differences between samples at a α = 0.05 confidence level. Capital letters indicate statistical difference compared to the control using the one-sample *t*-test, while lowercase letters indicate statistical differences between cookies enriched with protein within the two groups: samples without inulin (*n* = 18) and samples enriched with inulin (*n* = 23) according to the Tukey-HSD post hoc test. ^1^ The composition and abbreviations of the cookie samples are given in Table 2.

**Table 8:** Sensory properties of vegan lentil cookies.

Sample ^1^	Sweet Taste	Lentil Taste	Hardness	Crunchiness	Crumbliness
C	6.0 A	4.0 A	7.0 A	8.0 A	3.0 A
PS	5.1 ± 2.2 A,a	4.2 ± 2.1 A,a	5.8 ± 2.4 A,a	6.9 ± 2.2 A,ab	5.7 ± 3.0 B,b
BR	5.1 ± 2.4 A,a	5.0 ± 2.7 A,a	6.1 ± 2.5 A,a	6.4 ± 2.4 B,a	4.4 ± 2.7 B,ab
YP	4.4 ± 2.5 B,a	3.8 ± 3.0 A,a	8.8 ± 2.3 B,b	8.6 ± 1.9 A,b	2.9 ± 3.0 A,a
HS	5.9 ± 2.3 A,a	4.6 ± 2.9 A,a	5.1 ± 1.8 B,a	5.7 ± 2.1 B,a	5.4 ± 2.6 B,b
C-DF	6.0 A	4.0 A	7.0 A	8.0 A	3.0 A
PS-DF	5.3 ± 2.5 A,ab	3.9 ± 2.0 A,a	7.2 ± 2.5 A,b	7.0 ± 2.8 A,b	5.2 ± 3.0 B,b
BR-DF	5.7 ± 2.0 A,b	4.1 ± 2.3 A,a	7.5 ± 2.1 A,b	7.0 ± 2.3 A,b	4.6 ± 2.5 B,ab
YP-DF	4.0 ± 2.0 B,a	6.6 ± 3.0 B,b	3.5 ± 2.2 B,a	4.7 ± 1.8 B,a	5.4 ± 2.6 B,b
HS-DF	6.6 ± 2.4 A,b	4.1 ± 2.2 A,a	9.9 ± 0.3 B,c	9.8 ± 0.7 B,c	2.8 ± 3.6 A,a

Data presented as mean ± standard deviation. Within each column different capital letters and lowercase letters represent significant differences between samples at a α = 0.05 confidence level. Capital letters indicate statistical differences compared to the control using the one-sample *t*-test, while lowercase letters indicate statistical differences between cookies enriched with protein within the two groups: samples without inulin (*n* = 18) and samples enriched with inulin (*n* = 23) according to the Tukey-HSD post hoc test. ^1^ The composition and abbreviations of the cookie samples are given in Table 2.

## Data Availability

The original contributions presented in the study are included in the article/Supplementary Materials, further inquiries can be directed to the corresponding author.

## Supplementary Materials

The following supporting information can be downloaded at: https://www.mdpi.com/article/10.3390/foods14091518/s1, Table S1: Summary of Principal Components Analysis; Table S2: Variable importance.
