# Supplementary material for: Impact of Pumpkin Seed, Brown Rice, Yellow Pea, and Hemp Seed Proteins on the Physicochemical, Technological, and Sensory Properties of Green Lentil Cookies
Source: Foods. 2025 Apr 26;14(9):1518. doi: 10.3390/foods14091518 (PMC12071212; doi:10.3390/foods14091518)
Supplement: Supplementary file 1 [file foods-14-01518-s001.zip › Table S1.pdf]

Table S1. Summary of Principal Components Analysis

Component	R2X	R2X(Cumul.)	Eigenvalues	Q2	Limit	Q2(Cumul.)	Significance	Iterations
1	0,245621	0,245621	8,842358	-0,026740	0,135802	-0,026740	S	13
2	0,219579	0,465200	7,904827	0,046170	0,150000	0,020665	S	13
3	0,146555	0,611754	5,275965	-0,053121	0,168067	-0,031359	S	16
4	0,115862	0,727616	4,171029	-0,039386	0,191919	-0,071980	S	13

Number of components is 4. 72,7616% of sum of squares has been explained by all the extracted components.
