# Supplementary material for: Impact of Pumpkin Seed, Brown Rice, Yellow Pea, and Hemp Seed Proteins on the Physicochemical, Technological, and Sensory Properties of Green Lentil Cookies
Source: Foods. 2025 Apr 26;14(9):1518. doi: 10.3390/foods14091518 (PMC12071212; doi:10.3390/foods14091518)
Supplement: Supplementary file 1 [file foods-14-01518-s001.zip › Table S2.pdf]

Table S2. Variable importance

Variable	Variable number	Category value	Power	Importance
crunchiness	10		0,972036	1
hardness (sens)	9		0,971901	2
cohesiveness	14		0,969370	3
spread ratio	23		0,960636	4
height	21		0,954577	5
CP	27		0,948463	6
axTPC	24		0,944935	7
axCuprac	26		0,937621	8
baked odour	5		0,933342	9
sweet taste	8		0,927117	10
diameter	22		0,927076	11
hardness (text)	12		0,922567	12
sample code {YP}	1	114	0,897647	13
BI	19		0,887354	14
tea odour	4		0,874807	15
gumminess	15		0,872906	16
b*	18		0,806066	17
tea taste	7		0,785914	18
L*	16		0,782220	19
crumbliness	11		0,765255	20
adhesive force	13		0,728546	21
sample code {YP_DF}	1	119	0,709908	22
lentil taste	6		0,698569	23
axTFC	25		0,696683	24
a*	17		0,687813	25
surface hom.	2		0,674155	26
baking loss	20		0,654382	27
sample code {HE_DF}	1	120	0,536805	28
sample code {C}	1	111	0,476070	29
green (sens)	3		0,465456	30
sample code {HE}	1	115	0,454396	31
sample code {C_DF}	1	116	0,410936	32
sample code {BR}	1	113	0,370554	33
sample code {BR_DF}	1	118	0,343489	34
sample code {PS_DF}	1	117	0,123394	35
sample code {PS}	1	112	0,121213	36
